# Evaluating pedestrian crossing safety: Implementing and evaluating a convolutional neural network model trained on paired aerial and subjective perspective images

**DOI:** 10.1016/j.heliyon.2025.e42428

**Published:** 2025-02-03

**Authors:** Dylan Russon, Antoine Guennec, Juan Naredo-Turrado, Binbin Xu, Cédric Boussuge, Valérie Battaglia, Benoit Hiron, Emmanuel Lagarde

**Affiliations:** a University of Bordeaux, INSERM BPH U1219, Bordeaux, F-33000, France; b EuroMov Digital Health in Motion, Univ Montpellier, IMT Mines Ales, Ales, France; c CEREMA, France

**Keywords:** Pedestrian safety, Pedestrian crossings, Deep learning, Convolutional neural networks, Image segmentation, Infrastructure analysis

## Abstract

With pedestrian crossings implicated in a significant proportion of vehicle-pedestrian accidents and the French government's initiatives to improve pedestrian safety, there is a pressing need for efficient, large-scale evaluation of pedestrian crossings. This study proposes the deployment of advanced deep learning neural networks to automate the assessment of pedestrian crossings and roundabouts, leveraging aerial and street-level imagery sourced from Google Maps and Google Street View. Utilizing ConvNextV2, ResNet50, and ResNext50 models, we conducted a comprehensive analysis of pedestrian crossings across various urban and rural settings in France, focusing on nine identified risk factors.

Our methodology incorporates Mask R-CNN for precise segmentation and detection of zebra crossings and roundabouts, overcoming traditional data annotation challenges and extending coverage to underrepresented areas. The analysis reveals that the ConvNextV2 model, in particular, demonstrates superior performance across most tasks, despite challenges such as data imbalance and the complex nature of variables like visibility and parking proximity.

The findings highlight the potential of convolutional neural networks in improving pedestrian safety by enabling scalable and objective evaluations of crossings. The study underscores the necessity for continued dataset augmentation and methodological advancements to tackle identified challenges. Our research contributes to the broader field of road safety by demonstrating the feasibility and effectiveness of automated, image-based pedestrian crossing audits, paving the way for more informed and effective safety interventions.

## 1 Introduction

Pedestrian crossings account for 86% of all pedestrian accidents involving motorized vehicles, with two-thirds of these accidents occurring even when crossings are properly executed [1]. In response, the French government has undertaken initiatives to improve pedestrian safety, particularly by addressing the potential visibility issues between pedestrians and vehicles caused by infrastructure. Legislation introduced on May 22, 2020, prohibits motorized vehicles from parking within 5 meters of a pedestrian crossing, and mandates the removal of all such parking spaces by the end of 2026 [2], [3]. Moreover, in 2018 the French national road safety council gave a recommendation for an audit of the current state of France's pedestrian crossing infrastructure [4].

These safety measures face significant logistical challenges due to the vast number of pedestrian crossings in France, estimated to be several million. To address this issue, we propose the deployment of advanced deep learning neural networks for locating pedestrian crossings and assessing configurations affecting road safety through image analysis, using both aerial and ground-level images.

In the context of pedestrian safety at zebra crossings, two predominant risk factors, namely vehicle speed and driver-pedestrian visibility, can be significantly influenced by infrastructure [5], [6], [7]. In addition to infrastructural factors, driver behavior significantly influences pedestrian safety outcomes. Previous studies have investigated driver evasive actions to prevent pedestrian collisions in various urban and non-urban contexts [8], highlighting the importance of understanding driver responses to pedestrian crossings. In locations where vehicular and pedestrian routes intersect, regulating vehicle speed is essential both for crash prevention and for mitigating severity in the event of a collision.

Visibility between drivers and pedestrians can be hindered by both static and dynamic obstructions. Static elements include factors such as vegetation, built structures, trash containers, and notably, parked vehicles. Dynamic elements are vehicles that temporarily obscure pedestrians from the view of other drivers. For example, when a bus stops near a pedestrian crossing to let passengers off, it can block the view of pedestrians attempting to cross, and drivers overtaking the bus may not see them. Similarly, on roads with multiple lanes in the same direction, one vehicle can obscure pedestrians from drivers in adjacent lanes. Therefore, it is generally advised to avoid placing crossings on roads with more than one lane in the same direction. Additionally, parking near and upstream of a pedestrian crossing can cause similar visibility issues.

During a road crossing, pedestrians are exposed. Consequently, it is vital to minimize the crossing duration, and thus the distance where the pedestrian is in peril. This is of particular importance for elderly and disabled pedestrians who move slowly and are at heightened risk [9], [10], [11]. Road development guides [12] recommend a maximum pedestrian crossing distance of 12 meters at signal-controlled intersections and 8 meters at other types of crossings. For longer crossing distances, implementing refuge islands can provide intermediate safety zones for pedestrians. Two primary infrastructural interventions moderate traffic speed at crossings: road size reduction (for example, through the addition of a refuge island or pedestrian curb extension), and the incorporation of vertical deflection elements (such as a speed table) at the pedestrian crossing.

However, assessing and modifying the vast number of pedestrian crossings to address these safety concerns is a daunting task. To overcome this challenge, automated methods leveraging artificial intelligence (AI) and deep learning have been proposed for efficient analysis and assessment of road infrastructure.

In recent years, advancements in AI and deep learning have revolutionized various fields, including transportation research. Specifically, the application of computer vision techniques to road safety analysis has gained significant traction. Early efforts utilized convolutional neural networks (CNNs) for tasks related to autonomous driving, such as pedestrian detection and road segmentation [13], [14]. The increasing availability of robust machine learning libraries [15], [16] has facilitated the development of sophisticated models capable of analyzing complex road scenes.

Deep learning techniques have been employed in various aspects of transportation safety, including the detection of road damages [17], assessment of crash risks [18], traffic sign detection [19], and global road safety evaluation [20]. These advancements highlight the potential of AI in enhancing road safety by automating the analysis of vast amounts of visual data.

Segmentation, a fundamental task in computer vision, involves partitioning an image into semantically meaningful regions. This technique plays a pivotal role in various applications, from medical imaging to autonomous driving. In the realm of transportation safety, segmentation has garnered significant attention for its potential in identifying critical elements such as road infrastructure and pedestrian crossings.

Deep learning architectures, particularly those leveraging convolutional neural networks, have demonstrated remarkable performance in segmenting objects within complex scenes and in classification tasks.

Building upon these advancements, our study utilizes advanced deep learning models to analyze both aerial and ground-level images for locating pedestrian crossings and assessing configurations affecting road safety.

## 2 Methodology

In this methodology section, we present a detailed account of our approach, beginning with the training methodology for the zebra crossing detection model on aerial images from Google Maps, followed by the classification methodology for zebra crossings based on key safety variables.

### 2.1 Detection of zebra crossings & roundabouts

We leveraged the robust capabilities of Mask R-CNN [21] to train a segmentation model specifically tailored for zebra crossing detection. By exploiting aerial imagery obtained from Google Maps, we aimed to overcome the limitations of traditional annotation methods and extend coverage to regions not adequately represented in existing datasets.

In this subsection, we detail our methodology for training the segmentation model, encompassing data collection, preprocessing, model architecture selection, and training procedures. Through the utilization of Mask R-CNN, we seek to achieve accurate and scalable detection of zebra crossings, contributing to enhanced pedestrian safety analysis in diverse urban and rural environments.

#### 2.1.1 Segmentation approach

Segmentation is a fundamental task in computer vision that involves partitioning an image into semantically meaningful regions. This technique plays a pivotal role in various applications, from medical imaging to autonomous driving. In the realm of transportation safety, segmentation has garnered significant attention for its potential in identifying critical elements such as road infrastructure and pedestrian crossings.

Various approaches to segmentation have been explored in the literature, ranging from traditional methods like thresholding and region growing to advanced deep learning-based models. Deep learning architectures, particularly those leveraging convolutional neural networks (CNNs), have demonstrated remarkable performance in segmenting objects within complex scenes.

Several segmentation models exist, including U-Net [22], SegNet [23], and Fully Convolutional Networks (FCNs) [24]. U-Net is widely used for biomedical image segmentation due to its encoder-decoder architecture that captures context and enables precise localization. However, U-Net is primarily designed for pixel-wise semantic segmentation and may struggle with differentiating individual instances when multiple objects of the same class are present [25], [26]. SegNet, with its encoder-decoder architecture and pooling indices, is efficient in segmentation tasks but similarly focuses on semantic segmentation without instance differentiation. FCNs revolutionized segmentation by replacing fully connected layers in favor of purely convolutional ones, enabling the model to accept images of arbitrary size, yet they focus strictly on semantic segmentation rather than instance-level segmentation.

For our application, instance segmentation is crucial as we need to detect and segment individual zebra crossings and roundabouts, not just classify pixels.

One prominent architecture for instance segmentation tasks is Mask R-CNN (Region-based Convolutional Neural Network). Introduced by He et al. [21], Mask R-CNN extends the Faster R-CNN framework by incorporating a segmentation branch alongside the existing object detection components. This integration enables precise delineation of objects at the pixel level, facilitating accurate identification and localization at the instance level.

At its core, Mask R-CNN comprises three primary modules: the backbone network, the Region Proposal Network (RPN), and the mask head.

The backbone network, typically based on convolutional neural networks such as ResNet, serves as a feature extractor. It processes input images to extract high-level features essential for subsequent tasks. The use of a pre-trained backbone network, such as ResNet-50, facilitates transfer learning and enhances the model's ability to generalize across diverse datasets.

The Region Proposal Network (RPN) operates in parallel with the backbone network and generates region proposals for potential object instances within the input image. These proposals are refined and filtered based on their likelihood of containing objects of interest, enabling efficient localization of candidate objects.

Finally, the mask head is responsible for generating segmentation masks for each detected object instance. This component employs a fully convolutional network architecture to predict pixel-wise masks corresponding to the object boundaries. By leveraging spatial information and contextual cues, the mask head achieves precise delineation of object boundaries, facilitating accurate instance segmentation.

Mask R-CNN's modular architecture, incorporating components for object detection and pixel-level segmentation, facilitates seamless integration of both tasks within a unified framework. Furthermore, the availability of pre-trained models, such as Mask R-CNN with a ResNet-50 backbone, accelerates model development and enhances generalization across diverse datasets. Overall, the robustness, accuracy, and versatility of Mask R-CNN make it the ideal choice for zebra crossing detection in aerial imagery.

While several segmentation models exist, Mask R-CNN was chosen over alternatives such as U-Net, SegNet, and Fully Convolutional Networks (FCNs) due to its superior performance in instance segmentation tasks. Unlike traditional semantic segmentation models, which assign a single label to each pixel in an image, Mask R-CNN provides precise delineation of object boundaries, enabling instance-level segmentation. This capability is particularly advantageous in scenarios where multiple objects of the same class coexist within an image, as it allows for individual identification and segmentation. Other models like U-Net and SegNet are more suited for semantic segmentation and may not perform as well in distinguishing individual instances within overlapping or closely situated objects.

Therefore, Mask R-CNN was chosen over other architectures due to its superior performance in instance segmentation tasks, which aligns with the requirements of our study.

Through the utilization of Mask R-CNN, we seek to achieve accurate and scalable detection of zebra crossings and roundabouts, contributing to enhanced pedestrian safety analysis in diverse urban and rural environments.

#### 2.1.2 Data collection and processing

This section outlines how aerial images were collected and processed to enable both zebra crossing detection and roundabout analysis.

Aerial images were randomly obtained from cities across France, ensuring a diverse representation of urban, suburban, and rural settings. We utilized the Google Maps API, up to $200 in free monthly usage, to acquire high-resolution aerial imagery. Parameters such as zoom level and image size were chosen to balance spatial resolution and coverage, resulting in a dataset of 3,000 images.

We then visually identified images containing zebra crossings and used an online annotation tool, MyVision [27], [28], to create polygon masks for each crossing. Of the 2,456 annotated images, 1,256 formed the training set and 1,200 formed the test set. To strengthen model robustness, we applied several augmentation techniques: *spatial partitioning*, *zooming*, and *rotation*. Spatial partitioning splits larger images into smaller segments (retaining labels only when crossings are visible in that segment), while zooming and rotation simulate variations in perspective and orientation.

**Figure 1:**
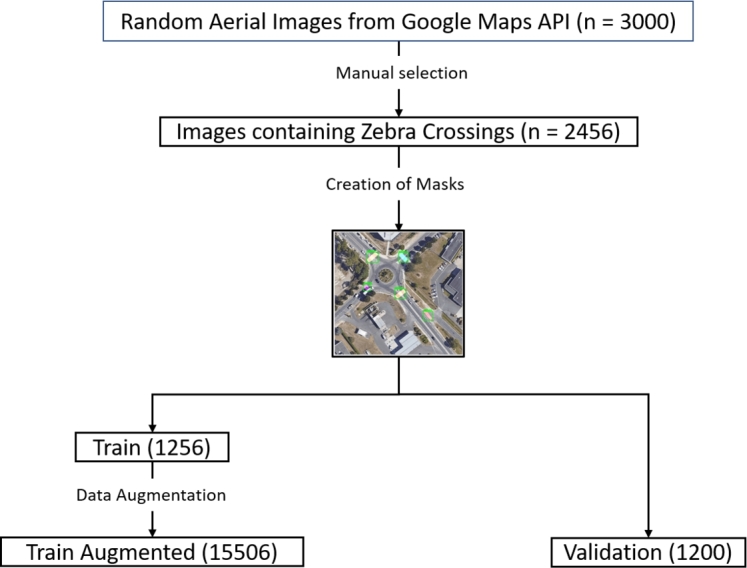
Data preparation for zebra crossing detection. This flowchart outlines the sequential steps taken, i.e., manual selection of relevant images, creation of masks, and data augmentation for the training dataset.

These steps increased the size of the training set to 15,506 augmented images, providing greater variation for robust model training (Fig. 1).

Roundabouts are a key factor in pedestrian safety, given their proximity to zebra crossings and potential safety risks. We therefore aimed to detect roundabouts in aerial imagery using a similar methodology as for zebra crossings. Specifically, we selected 773 aerial images containing roundabouts from diverse French regions (urban, suburban, and rural), allocating 400 to the validation set and 373 to the training set.

To comprehensively capture roundabouts and their context, each roundabout was annotated with two labels:

• **rd_int**: The interior of the roundabout, enabling accurate measurement of the roundabout's diameter.
• **rd_ext**: The roundabout and its connecting roads, allowing us to analyze spatial relationships between the roundabout and nearby infrastructure.

**Figure 2:**
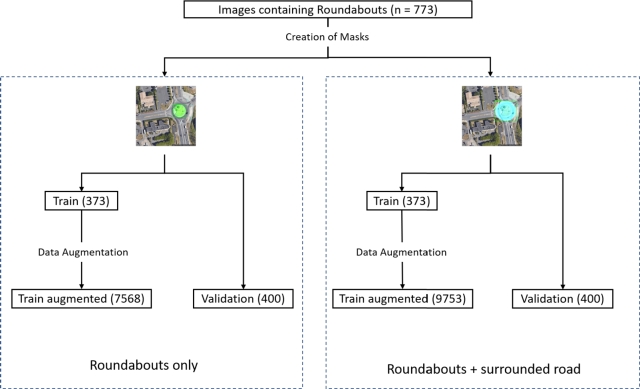
Data preparation for roundabout detection. The left flowchart illustrates the augmentation of aerial images of roundabouts only, while the right flowchart depicts the augmentation process for roundabouts with surrounding roads. Both processes begin with manual image selection, followed by the creation of masks and further data augmentation for the training datasets.

Having both labels is crucial for determining distances between roundabouts and zebra crossings, as any crossing within 5 meters of a roundabout poses higher safety risks due to limited driver reaction time and elevated vehicle speeds. The same augmentation methods used for zebra crossings (spatial partitioning, zooming, rotation) were applied here, ultimately increasing the training dataset to 9,753 images for roundabouts with surrounding roads (*rd_ext*) and 7,568 for roundabouts alone (*rd_int*). This expanded dataset provides a solid foundation for both roundabout detection and analysis (Fig. 2).

#### 2.1.3 Evaluation metrics

For evaluating the performance of our segmentation model, we employed the CocoEvaluator metric, which has emerged as the state-of-the-art for segmentation evaluation metrics. The COCO (Common Objects in Context) evaluation metrics provide a comprehensive framework for assessing the accuracy of object detection and segmentation models. These metrics, including Average Precision (AP) and Average Recall (AR) across different IoU (Intersection over Union) thresholds, offer detailed insights into the model's performance in various aspects of the segmentation task [29].

The choice of CocoEvaluator is motivated by its widespread adoption in the research community, providing a standardized benchmark for comparing the performance of different segmentation models. This metric allows us to assess not only the accuracy of our model in detecting zebra crossings and roundabouts but also the precision of the segmentation boundaries, which is crucial for subsequent analysis and application in pedestrian safety systems.

#### 2.1.4 Training procedure

The Mask R-CNN model was trained using standard hyperparameters commonly recommended in the literature and defaults provided by deep learning frameworks. Each training session consisted of 10 epochs, allowing the model to learn effectively without overfitting to the training data.

We adopted a cosine learning rate scheduler to adjust the learning rate throughout the training process, starting from an initial learning rate of 0.05. A momentum of 0.9 was employed to accelerate gradient descent in relevant directions, while a weight decay of 0.0005 helped prevent overfitting by penalizing large parameter values.

The batch size for training was set to 4 per device, utilizing a total of 2 NVIDIA GeForce RTX 3090 Ti GPUs for parallel processing, resulting in a total training batch size of 8. This configuration efficiently utilized available computational resources while maintaining stability during training. Similarly, a test batch size of 4 per device was employed during evaluation to ensure consistent performance metrics.

### 2.2 Classification methodology for zebra crossings

In this section, we will explain the methodology used to train our classification models for pedestrian crossing safety assessment.

#### 2.2.1 Selection of key variables for pedestrian crossing safety assessment

The variables selected for assessing pedestrian crossing safety in this study include: presence of trapezoidal speed bumps or plateaus, visibility issues (e.g., obstructions blocking the view between pedestrians and drivers), presence of an advanced stop line or cyclist waiting area at traffic lights, presence of public lighting within 20 meters, crossing length, presence of parking spaces within 5 meters upstream of the pedestrian crossing, presence of a dangerous refuge island (crossable or large size smaller than 2 meters), presence of a bus stop upstream of the pedestrian crossing, presence of a tactile warning strip.

**Figure 3:**
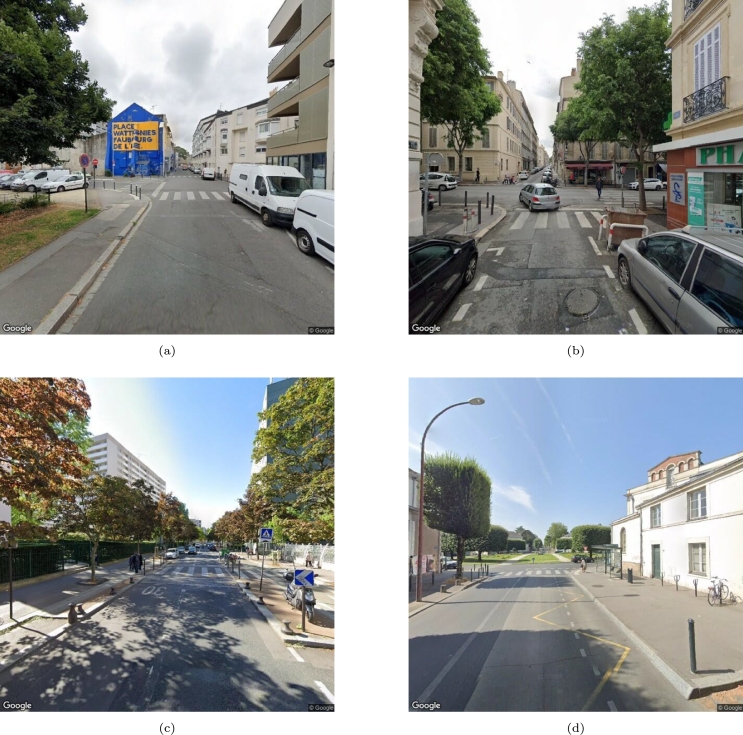
Examples of problematic zebra crossings. (a) & (b): Lack of visibility between pedestrians crossing the road and oncoming vehicles due to the parked van and the trash container. (c): A curb extension that creates a protective area around the crossing, preventing situations like (a) and (b). (d): A potentially dangerous bus stop. A bus stopped in the marked region can hide a pedestrian crossing the road from overtaking vehicles [30].

These variables were chosen based on recommendations from road development guides and previous research, owing to their direct influence on factors like vehicle speed, driver-pedestrian visibility, and pedestrian exposure during crossing. Examples of problematic zebra crossings are shown in Fig. 3.

#### 2.2.2 Data collection and processing: developing a customized image dataset of pedestrian crossings

Our image dataset of pedestrian crossings was compiled by integrating information from two primary sources: Google Maps (GM) and Google Street View (GSV). Each observation is composed of a pair of images: an aerial view sourced from GM and a street-level view obtained from GSV.

The initial version of this dataset was constructed using OpenStreetMap (OSM) data to obtain geospatial coordinates of zebra crossings in major French cities. Our initial focus was on collecting street-level images from Google Street View in the 11 most populous cities in France—including Paris, Marseilles, Lyon, Toulouse, Nice, Nantes, Montpellier, Strasbourg, Bordeaux, Lille, and Rennes—to analyze pedestrian crossings in urban environments. This dataset was intended for training classification models to assess various safety-related attributes of pedestrian crossings. Using OSM data, we constructed a dataset encompassing 37,835 zebra crossings.

However, we recognized that relying solely on OSM data from major cities limited the diversity of our dataset, particularly in representing crossings in smaller towns or rural areas where certain configurations (e.g., lack of infrastructure, different traffic conditions) are more common. To address this, we expanded our dataset by leveraging aerial images obtained via the Google Maps API, as Google's extensive geographic coverage includes a broader range of regions in France. We then applied our trained segmentation model to these aerial images from various cities and towns across the country to detect additional zebra crossings, especially in locations where OSM data was sparse or incomplete. This allowed us to collect a more diverse set of zebra crossings, including those from underrepresented regions.

Additionally, we decided to incorporate aerial images of the zebra crossings into our model alongside the street-level images. Incorporating aerial images provided additional spatial context and allowed the models to capture features not easily visible from street-level images alone. Aerial views were instrumental in assessing the presence of speed bumps, parking spaces near crossings, configurations of refuge islands, and bus stops. The combination of aerial and street-level perspectives enriched the dataset, enabling the models to learn more comprehensive representations of the crossing environments, which in turn enhanced classification performance.

To combine both aerial and street-level images for each zebra crossing, we follow a straightforward fusion approach. Specifically, we concatenate the aerial and street-level images side by side, resulting in a single input image of size 1280×640 pixels (each image is 640×640). This fused image is then fed into the neural network, allowing it to learn from both the global context provided by the aerial perspective and the detailed features seen at street level in a single forward pass. We apply standard normalization steps to the concatenated image, but no additional augmentation is performed to preserve each perspective's unique visual information.

To evaluate the impact of incorporating aerial images, we conducted an experiment comparing the performance of models trained using only street-level images versus models trained using both aerial and street-level images concatenated as input. The results indicated that models utilizing both perspectives outperformed those using only street-level images across several key variables (see Appendix A). This suggests that the additional information provided by aerial views is valuable for the accurate classification of features affecting pedestrian safety.

For example, in the detection of speed bumps, models trained with both aerial and street-level images achieved higher PRC-AUC scores compared to those trained with street-level images alone. This improvement can be attributed to the aerial images' ability to provide a clear view of road surface markings and structures that are not always visible or distinguishable from the street-level perspective.

These findings underscore the importance of multi-perspective data in enhancing the models' ability to accurately identify and assess safety-related features of pedestrian crossings.

To acquire a more diverse set of zebra crossing images using the Google Maps API, we developed a custom software application that streamlines the selection and extraction process of image pairs for any specified area. We employed a stratified random sampling technique, selecting towns based on their population size categories. From each selected town, we extracted up to 200 aerial images, or fewer if not as many were available. In the first step, the postal code of the relevant area was inputted. The application subsequently employed the Mask R-CNN trained model to analyze the aerial views of the selected area, aiming to identify pedestrian crossings.

This method added an additional 11,526 couple of zebra crossing images from various towns, enriching our dataset with scenarios that may not be encountered in larger cities.

For each pedestrian crossing identified, we extracted its coordinates, geographical orientation, and metric characteristics (length and width). These parameters were used to delineate the 640×640 formatted aerial image for further use.

To integrate Google Maps (GM) data with Google Street View (GSV) data, we followed a detailed process:

1. **Determining Street View Parameters:** Using the coordinates of each detected crossing, we calculated the optimal position and orientation for capturing the street-level image from GSV.
  • **Positioning:** Based on the zebra crossing coordinates and the location of the road, new coordinates points were obtained to be placed at approximately 15 meters upstream from the crossing along the direction of traffic, simulating the viewpoint of an approaching driver.
  • **Orientation (Heading):** The Heading was obtained to calculating the nearest point on the road so that the image faced directly towards the crossing to ensure it was prominently visible.
  • **Pitch:** The vertical angle was set to 0 degrees for a horizontal view.
2. **Extracting Street View Images:** We retrieved a 640×640 pixel street-level image from GSV for each crossing using the calculated parameters, utilizing the Google Street View Static API.
3. **Creating Paired Images:** The GM aerial image and the GSV street-level image were concatenated side by side to form a combined image of size 1280×640 pixels. This approach allows the model to analyze both perspectives simultaneously.
4. **Data Preparation:** The concatenated images were normalized according to the input requirements of the neural network models. No additional preprocessing beyond standard scaling was applied.

By providing both aerial and street-level views in a single input image, the models can leverage complementary information from each perspective. The aerial images offer a broader context of the crossing's surroundings, while the street-level images provide detailed visual information about the crossing's features and potential obstacles.

**Figure 4:**
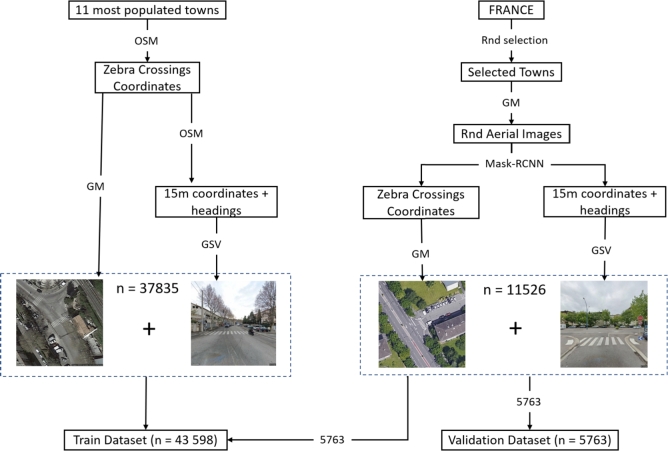
The two-part dataset preparation process for the classification of pedestrian crossings. The left branch represents the initial data collection strategy, primarily utilizing OpenStreetMap (OSM) and Google Street View (GSV) to focus on street-level images, predominantly from major urban centers. This resulted in a dataset size of 37,835 images. The right branch introduces the revised methodology, which uses the Mask R-CNN model trained to detect zebra crossings to include diverse locations across France. This enhancement expanded the dataset to include 11,526 images that capture a more representative sample of pedestrian crossings nationwide. In both branches, each aerial and street-level image pair is concatenated horizontally, producing a single 1280 × 640 image for the classification models.

To further enhance the representation of our dataset, we revised our dataset split to include the newly acquired images from diverse towns. The first 37,835 pairs of zebra crossing images, primarily from the major cities, were allocated to the training dataset. The new images obtained through our expanded data collection method were randomly split into two groups. Half of these image pairs (5,763) were added to our training dataset, bringing the total to 43,598 training image pairs. The remaining 5,763 image pairs were allocated to our evaluation dataset. This strategic distribution ensures that our model is trained on a diverse set of scenarios, encompassing both urban and rural environments, and that our evaluation metrics are reflective of the model's performance across any town in France (Fig. 4).

#### 2.2.3 Labeling the dataset

Our labeling evaluated the presence and absence of different safety elements, conformally with our risk assessment described above. To maintain stability and limit annotation subjectivity, we set each label to be boolean (i.e., True or False only). In refining our labeling process, we recognized that certain attributes of pedestrian crossings could be more accurately and efficiently determined through automated methods rather than manual annotation. Specifically, the length of pedestrian crossings, initially a part of our manual labeling process, was found to be directly calculable from the aerial images. By measuring these dimensions algorithmically, we eliminated the need for manual labeling of this feature, thereby increasing accuracy and reducing the potential for human error.

Furthermore, for the assessment of dangerous refuge islands, we adopted a more sophisticated approach. We used the ResNet-based Mask R-CNN model trained to detect and segment roundabouts in aerial imagery. Following the detection, a subsequent analysis was conducted on these roundabouts, examining factors such as the size of the roundabout, the distance to nearby pedestrian crossings, and their relative positions. This allowed us to ascertain whether a pedestrian crossing near a roundabout could be classified as ‘dangerous’ based on these objective criteria. This method not only streamlined the labeling process but also introduced a higher level of precision in identifying potentially hazardous pedestrian crossings near roundabouts.

We annotated the following elements for each pair of images:

1. Valid pedestrian crossing: The image is of a pedestrian crossing and we see at least the crossing from two safe areas (either a side of the road or a refuge island). Moreover, the crossing should be with zebra markings and in correct condition. This insures stability for the analysis of the images by the neural network.
2. Speed bump plateau: Indicate if there is a speedbump plateau.
3. Lack of visibility: Indicate if there is a object, outside of usual traffic, blocking the view of the pedestrian crossing.
4. Bad parking spot: Indicate if there is a parking space in the 5 meters before the start of the zebra crossing.
5. Traffic light: Specify the presence of a traffic light at the crossing
6. Advanced Stop Line at traffic light: Mark the presence of a advanced stop line at the traffic light, at least 5 meters before the crossing. This ensures cars have a wide view on the crossing before starting when the light turns green.
7. Refuge Island: Indicate if there is a refuge island
8. Dangerous Refuge Island: In the case that there is a refuge island, we check if it protects pedestrians correctly, i.e. it is sufficiently large and high.
9. Bus Stop proximity: Specify if the pedestrian crossing is located near a roundabout in a non-secure manner. This is the case if the pedestrian crossing is set continuously (i.e. without passing through a refuge island) across two lanes or if it is too close.

#### 2.2.4 Addressing data imbalance

A significant challenge in training our models was the imbalance in the dataset across different classes. Certain key variables, such as the presence of speed bumps or bus stops near crossings, had significantly fewer positive instances compared to negative ones. This imbalance can lead to models that are biased toward the majority class, reducing their ability to accurately detect underrepresented features.

To address the class imbalance without altering the datasets or employing extensive data augmentation techniques, we focused on using evaluation metrics that are appropriate and informative for imbalanced data scenarios. Relying on accuracy alone can be misleading in the presence of class imbalance, as a model could achieve high accuracy by simply predicting the majority class. Instead, we utilized metrics such as:

• **F1-Score**: Provides a balance between precision and recall, offering a better measure of the incorrectly classified cases than accuracy.
• **Precision-Recall Curve Area Under the Curve (PRC-AUC)**: Offers insights into the trade-off between precision and recall for different threshold settings, particularly useful for imbalanced datasets.
• **Receiver Operating Characteristic Area Under the Curve (ROC-AUC)**: Measures the ability of the model to distinguish between classes across all threshold settings.

**Figure C.16:**
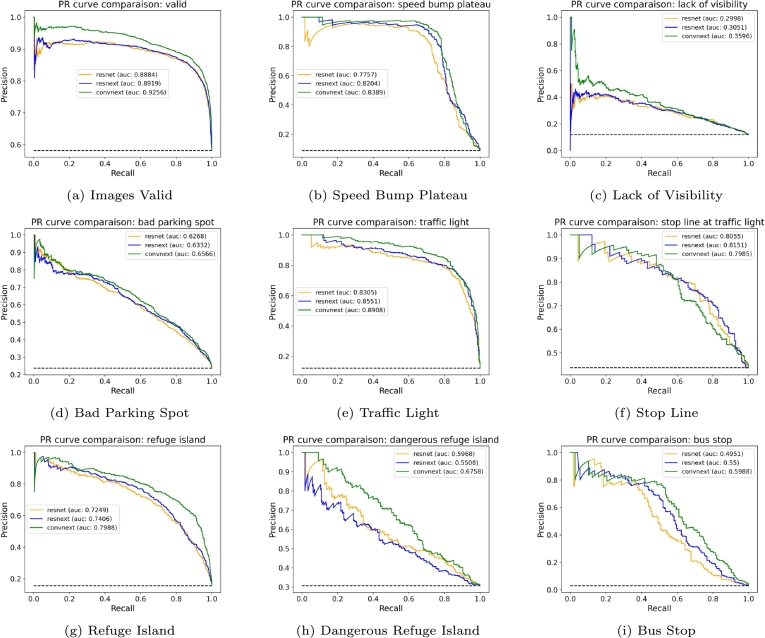
Precision-Recall curve by Threshold comparison for each key variables. The dashed horizontal line in each plot indicates the naive classifier performance, computed as the fraction of positive instances in the training set for that variable.

Furthermore, we included naive baseline performance in our Precision-Recall curves (e.g., Fig. 9 and Appendix C Fig. C.16). The naive baseline represents the performance of a classifier that makes predictions based solely on the prevalence of the positive class in the dataset. Specifically, it is calculated by taking the ratio of positive instances to the total number of instances, effectively serving as a reference point. In our plots, the dashed lines represent this naive baseline, demonstrating that our models outperform these baselines despite the class imbalance.

By evaluating the models using these metrics and comparing them against the naive baseline, we could more accurately assess their performance in detecting the minority classes (positive instances) despite the imbalance. Additionally, during model training, we used appropriate loss functions, such as binary cross-entropy loss, which can be adjusted to account for class imbalance by weighting the loss associated with each class differently. This approach helps to mitigate the impact of class imbalance on the training process.

#### 2.2.5 A comparison of three different neural networks

We initially chose to use ResNet-50 [31] for our neural network architecture, as it is among the best for image classification and has largely proven its worth in the AI community as a baseline for image classification. It is a 50-layer residual network with approximately 25.5 million trainable parameters. This relatively low number of parameters^1^ allows it to run on limited equipment, and it offers a great balance between performance and computational efficiency. Finally, this model is widely available pre-trained on the ImageNet dataset [32] in most deep learning libraries. This pre-training is key, as training the neural network from scratch on just our dataset yields worse results and requires a much longer time to train. Pre-training on a large image database such as ImageNet (approximately 1,300,000 images) allows the model to pick up general image analysis features that it cannot acquire otherwise from just a small dataset like ours (40,000 pairs of images). For each label, we trained a separate neural network and used the binary cross-entropy as our loss function for gradient descent.

In our pursuit of optimizing the performance of our neural network models for pedestrian crossing analysis, we extended our exploration beyond the initially chosen ResNet-50 architecture. Recognizing the advancements in neural network designs, we included two additional models in our comparative study: ResNeXt50 and ConvNeXt. These models were selected due to their promising capabilities in image recognition tasks, which had not been available during the initial phases of our research.

ResNeXt50 [33], an advanced version of the classic ResNet architecture, introduces a novel concept in deep learning known as ‘cardinality’. This concept refers to the number of parallel paths within a ResNet block. Unlike the traditional ResNet block, which has a single path, ResNext50 employs multiple paths of convolutional layers, each with its own set of filters. This structure is akin to having an ensemble of layers working in tandem within each block, allowing the network to learn a rich and diverse set of features. Despite its complexity, the architectural design of ResNeXt50 maintains computational efficiency by distributing its filters across these multiple paths. Furthermore, the model retains the bottleneck design from ResNet, which is instrumental in reducing computational overhead. The ResNeXt50 architecture is particularly effective in tasks requiring the recognition of various aspects of an image, as seen in complex image classification challenges, due to its capability to learn a wide range of features.

ConvNext [34], standing for Convolutional Networks Next, represents a significant evolution in the design of convolutional neural networks. Drawing inspiration from the success of transformer models in handling sequential data, ConvNext adapts several key features from this domain. One of the prominent changes in ConvNext is the use of layer normalization instead of the commonly used batch normalization found in traditional CNNs. Layer normalization adjusts the inputs across each channel in a layer, leading to improved training stability and model performance. Additionally, ConvNext employs the Gaussian Error Linear Unit (GELU) activation function, a smoother and more effective alternative to the widely used ReLU function. This activation function contributes to the model's ability to capture more complex patterns in data. Another critical aspect of ConvNext is its utilization of depthwise separable convolutions, which significantly reduce the number of parameters and computational costs, thereby enhancing the efficiency of the network. ConvNext's design makes it well-suited for a variety of computer vision tasks, offering advantages in terms of computational efficiency and the capacity to capture intricate patterns in imagery.

We trained each of the three models by concatenating the pair of images as input. For each key variable, we trained a separate binary classification model. Each model was trained to predict the presence or absence of a specific safety-related feature at pedestrian crossings. This approach allows each model to specialize in recognizing patterns relevant to its assigned feature, potentially improving overall accuracy and performance.

**Figure B.11:**
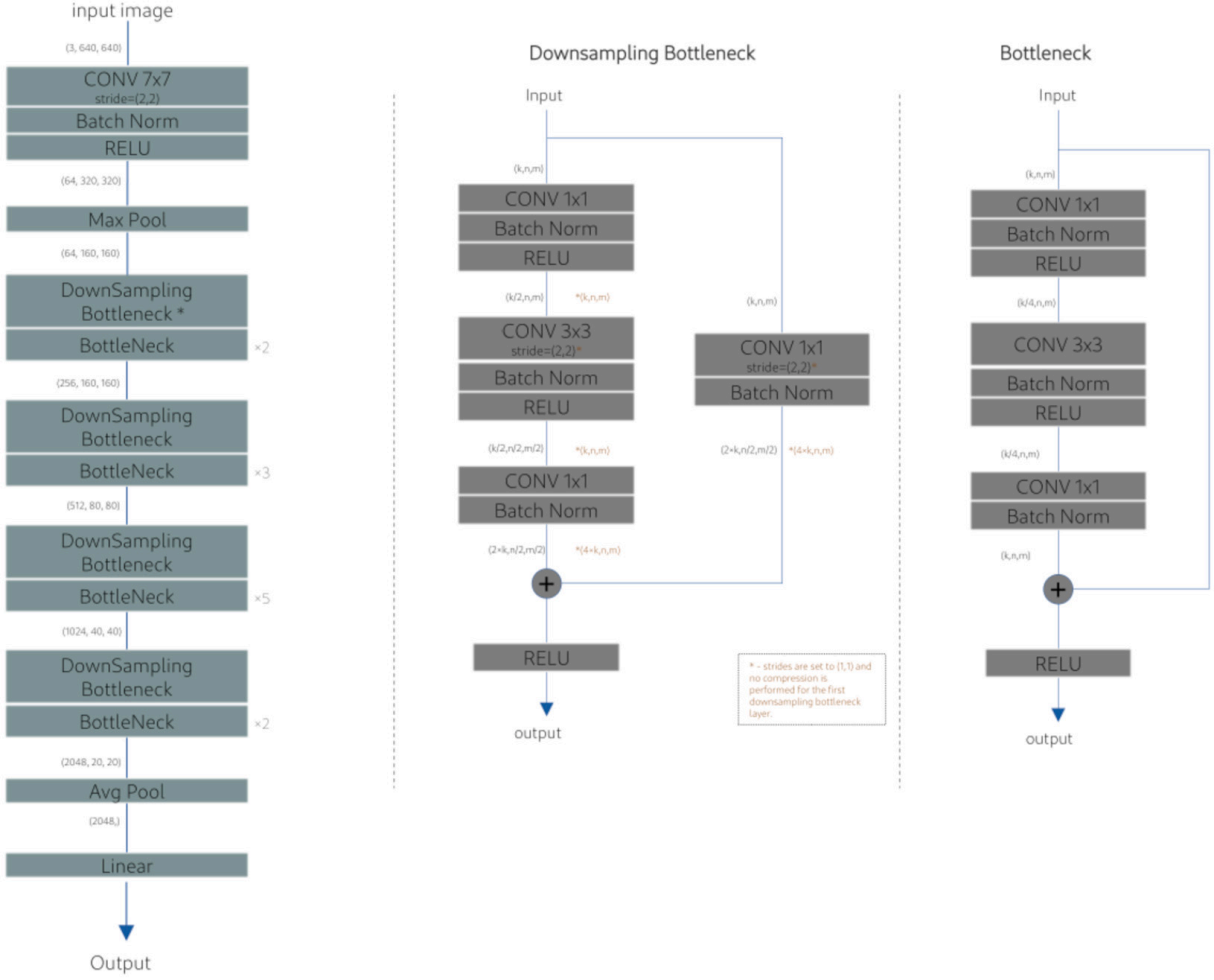
The ResNet50 architecture. The ResNet50 neural network is composed of 49 convolutional layers (not counting the downsampling convolution) and 1 linear layer. The main component of the ResNet model is the bottleneck module that does two things at the same time: it compresses and decompresses the input information, and adds a side connection where the original information is maintained.

**Figure B.12:**
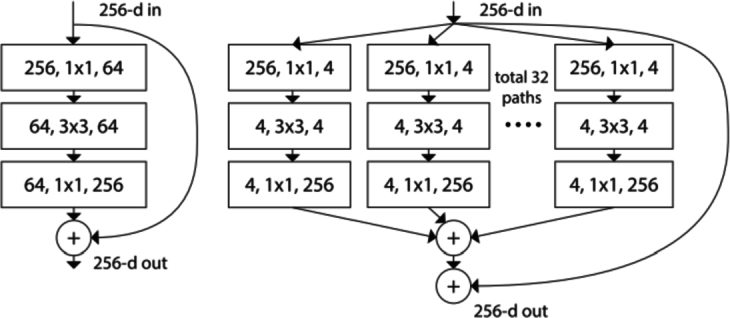
ResNet and ResNext difference. This image compares the block structure of a ResNet module (left) with a ResNeXt module (right). The key difference is the introduction of cardinality in ResNeXt, which introduces multiple parallel convolutional paths within each block. The ResNeXt block aggregates these multiple paths, providing richer feature representation and enhancing the model's capacity to capture complex patterns. Source image: [33].

**Figure B.13:**
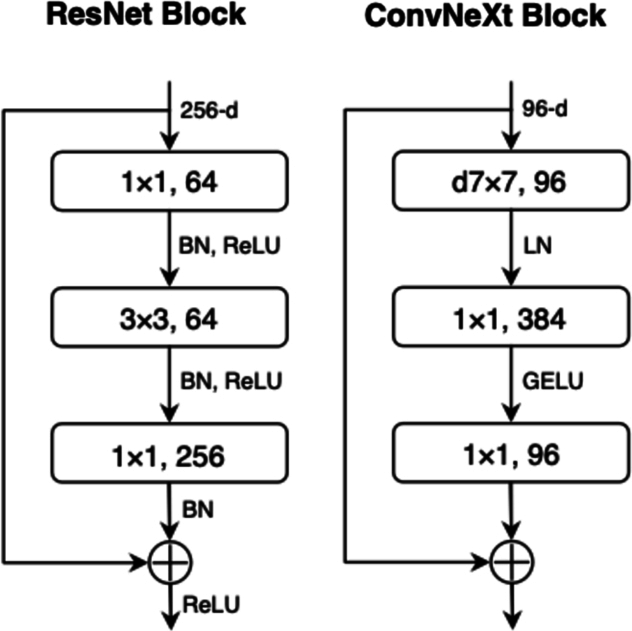
ResNet comparison to ConVNeXt. This image compares a ResNet block (left) with a ConvNeXt block (right). ConvNeXt replaces the traditional Batch Normalization and ReLU activation functions of ResNet with Layer Normalization (LN) and GELU activation, respectively. It also introduces a larger convolutional kernel size, which allows the network to capture broader context from images, enhancing the model's ability to learn complex image features. Source image: [34].

To aid in understanding these architectures, we have included schematic diagrams of ResNet-50, ResNeXt50, and ConvNeXt in Appendix B (Figure B.11, Figure B.12, Figure B.13 respectively). These figures illustrate the key components and differences of each model, providing a visual reference to complement our descriptions.

## 3 Results

### 3.1 Detection of zebra crossings & roundabouts

#### 3.1.1 Detection of zebra crossings

*Loss analysis*

**Figure 5:**
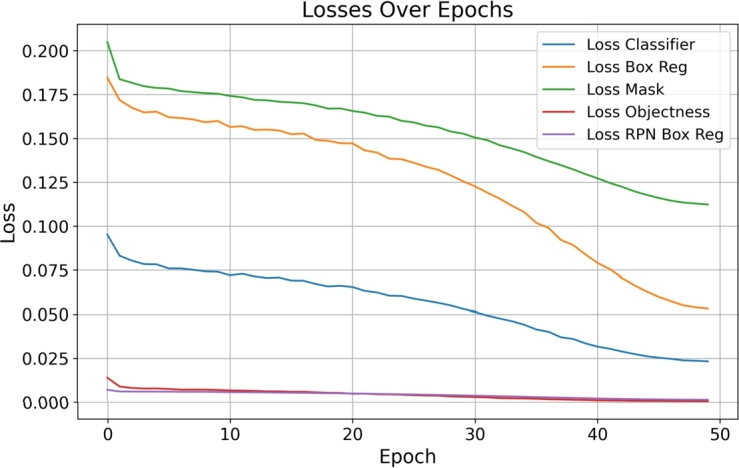
Loss Metrics Over Epochs. The figure illustrates the decrease in various loss components, including classifier loss, box regression loss, mask loss, objectness loss, and RPN box regression loss, as the model learns.

The losses incurred during the model's training are plotted in Fig. 5. The trends indicate a decrease in losses across all categories, suggesting that the model is effectively learning to detect and segment zebra crossings from aerial images (Fig. 5).

*Average precision and recall*

The performance of our Mask R-CNN model in the detection of zebra crossings was evaluated using various metrics, with a particular focus on Average Precision (AP) and Average Recall (AR) across different Intersection over Union (IoU) thresholds. These metrics are pivotal in understanding the model's accuracy and reliability in identifying and segmenting zebra crossings from aerial imagery.

**Figure 6:**
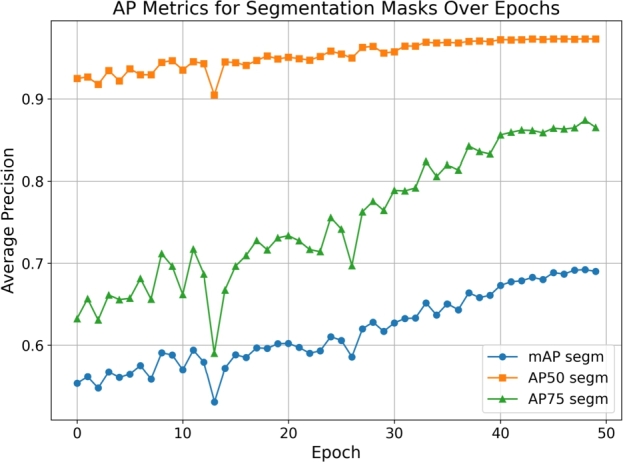
AP metrics for segmentation masks over epochs.

**Figure 7:**
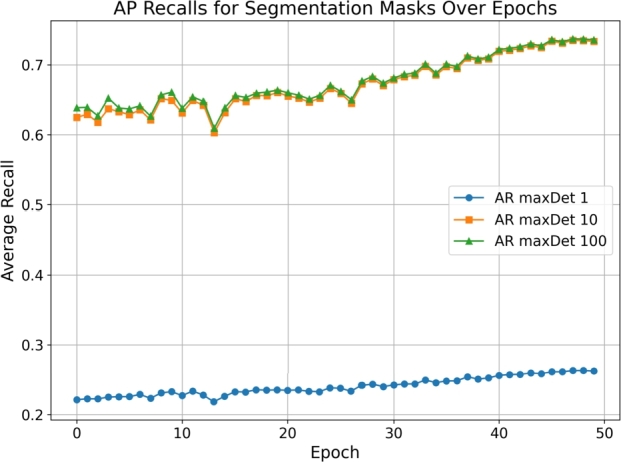
AR metrics for segmentation masks over epochs.

Fig. 6 displays the progression of AP metrics for segmentation masks over the training epochs. Notably, AP50 shows the model's ability to detect zebra crossings with moderate overlap, while AP75 illustrates detection with stricter overlap criteria. Fig. 7 presents the evolution of AR metrics over the epochs. The model's recall for detecting zebra crossings improves consistently, indicating an increasing ability to identify relevant instances within the dataset.

The model achieved an AP@[IoU:0.50:0.95] of 74.49%, which is a comprehensive metric representing the average precision across IoU thresholds from 0.5 to 0.95 (inclusive) with a step size of 0.05. At specific IoU thresholds, the AP50 reached 97.25%, indicating a high precision in detecting zebra crossings with a moderate overlap criterion. Meanwhile, the AP75 of 88.15% suggests that the model maintains a commendable precision even with a stricter overlap criterion.

Comparing these results to benchmarks in the domain, our model demonstrates competitive performance. For instance, He et al. [21] reported an AP@[IoU:0.50:0.95] of 63.1% for the Mask R-CNN model on the COCO dataset, highlighting the effectiveness of our tailored approach for zebra crossing detection in achieving superior precision.

The model's average recall for the top 100 detections (AR@[maxDets:100]) was 76.58% across all IoU thresholds, which is indicative of the model's ability to identify most of the zebra crossings. (See Table 1.)

**Table 1:** Summary of model performance metrics at last epoch.

Metric	Zebra Crossings	Roundabouts	Roundabouts + Roads
AP@[IoU:0.50:0.95-area:all]	74.49%	85.46%	74.62%
AP@[IoU:0.50-area:all]	97.25%	91.53%	81.61%
AP@[IoU:0.75-area:all]	88.15%	90.62%	77.76%
AP@[IoU:0.50:0.95-area:small]	62.84%	79.16%	40.50%
AP@[IoU:0.50:0.95-area:medium]	78.52%	85.76%	58.51%
AP@[IoU:0.50:0.95-area:large]	59.80%	90.34%	89.25%
AR@[IoU:0.50:0.95-maxDets:1]	28.42%	87.71%	78.98%
AR@[IoU:0.50:0.95-maxDets:10]	79.21%	88.31%	79.84%
AR@[IoU:0.50:0.95-maxDets:100]	76.58%	88.31%	79.84%
AR@[IoU:0.50:0.95-area:small]	70.23%	-	-
AR@[IoU:0.50:0.95-area:medium]	77.14%	88.61%	70.18%
AR@[IoU:0.50:0.95-area:large]	63.33%	91.52%	90.82%

#### 3.1.2 Detection of roundabouts

The performance of the Mask R-CNN model for roundabout detection was evaluated using two separate datasets: one for roundabouts only (*roundabout_int*) and another for roundabouts with surrounding roads (*roundabout_ext*). The training and validation datasets consisted of 773 images, which were augmented to include over 17,000 images through various techniques, ensuring robustness and generalizability of the model.

*Loss analysis*

Throughout the training epochs, the total loss exhibited a decreasing trend, indicating that the model was learning and improving its ability to segment the roundabouts in aerial images. The final epoch's total loss was reduced to 0.10, from an initial loss of 0.62, reflecting successful model convergence.

*Roundabouts only (roundabout_int)*

For the roundabouts only dataset, the model achieved a mean Average Precision (mAP) across all Intersection over Union (IoU) thresholds of 85.46% in the last epoch, showing substantial capability to identify roundabout features accurately. The AP at IoU threshold of 0.50 (AP50) was 91.53%, indicating high precision at less strict overlap criteria, while the AP at IoU threshold of 0.75 (AP75) was 90.62%, representing stricter criteria. The Average Recall (AR) for the model considering the top 100 detections (AR@100) reached 88.31%, showing that the model could detect the majority of positive roundabout instances present in the test dataset.

*Roundabouts with surrounding roads (roundabout_ext)*

Similarly, for the roundabouts with surrounding roads, the model demonstrated a strong performance with a mAP of 74.62%, an AP50 of 81.61%, and an AP75 of 77.76%, closely aligning with the performance on the roundabouts only dataset. The AR@100 for this dataset was also high at 79.84%.

**Figure 8:**
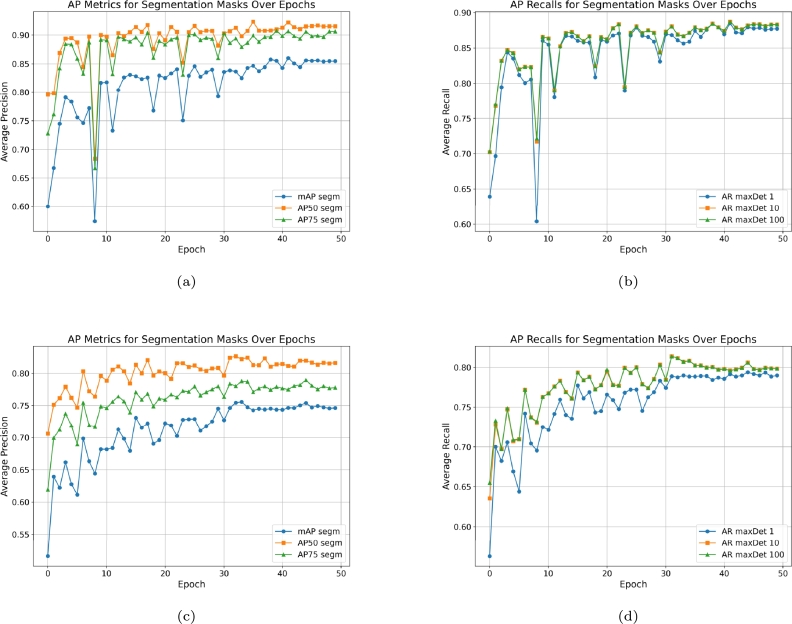
AP and AR metrics for segmentation masks over epochs for the roundabouts ((a) and (b)) and roundabouts + surrounding roads ((c) and (d)).

The consistent performance across both datasets signifies the model's effectiveness in capturing the necessary features for roundabout detection, regardless of the inclusion of surrounding road details (Fig. 8). The data also shows that there is a minimal drop-off in precision when moving from AP50 to AP75, which suggests that the model is relatively robust to the strictness of the overlap criteria.

*Comparative analysis*

When compared to other state-of-the-art models, the performance metrics indicate that the employed Mask R-CNN architecture provides competitive results for the task of roundabout detection in aerial imagery. These results corroborate the findings from similar studies, reinforcing the reliability of Mask R-CNN for complex segmentation tasks within the domain of transportation safety.

### 3.2 Classification of zebra crossings

Our study evaluated the performance of three distinct neural network architectures—ResNet50, ResNeXt50, and ConvNeXtV2—in the classification of zebra crossings based on various safety-related variables. These variables include the presence of speed bumps, visibility issues, parking spots, traffic lights, stop lines at traffic lights, refuge islands, dangerous refuge islands, and bus stops. We utilized metrics such as loss, F1 score, Precision-Recall Curve Area Under the Curve (PRC-AUC), Receiver Operating Characteristic Area Under the Curve (ROC-AUC), and Positive Weight Index (PWI) for our analysis. The dataset size varied across tasks, with a notable division between positive and negative labels, highlighting the challenges in achieving balanced datasets for certain variables (Table 2). To facilitate deeper insight into model predictions, we have included confusion matrices for selected tasks in Appendix D. These matrices display true positives, false positives, false negatives, and true negatives, offering a raw depiction of model performance for underrepresented labels.

**Table 2:** Training dataset distribution for each key variable.

Task	Dataset Size	Positive	Negative	PWI
Valid	43,315	29,267	14,048	0.48
Speed bump plateau	29,440	1,379	28,061	20.35
Lack of visibility	28,840	5,277	23,563	4.47
Bad parking spot	30,022	8,396	21,626	2.58
Traffic light	29,194	6,087	23,107	3.80
Stop line at the traffic light	5,841	2,284	3,557	1.56
Refuge island	30,686	5,819	24,867	4.27
Dangerous refuge island	5,601	1,678	3,923	2.34
Bus stop	30,875	731	30,144	41.24

#### 3.2.1 Performance overview

Table 3 summarizes the metrics (loss, F1-score, PRC-auc and ROC-auc) for each model and each variable. The models demonstrated variable performance across different tasks, with ConvNextV2 generally outperforming ResNet50 and ResNext50 in terms of PRC-AUC and ROC-AUC scores. For instance, in the task of detecting the presence of valid zebra crossings, ConvNextV2 achieved a PRC-AUC of 0.9256 and a ROC-AUC of 0.9191, compared to ResNet50's 0.8884 and 0.8906, and ResNext50's 0.8919 and 0.8930, respectively. This indicates a higher capability of ConvNextV2 in distinguishing between positive and negative instances with greater reliability.

**Table 3:** Comparison results for ResNet50, ResNeXt50, and ConvNeXtV2 for each key variable on loss, F1 score, PRC-AUC, and ROC-AUC.

Task	ResNet50				ResNeXt50				ConvNeXtV2			
	Loss	F1	PRC-AUC	ROC-AUC	Loss	F1	PRC-AUC	ROC-AUC	Loss	F1	PRC-AUC	ROC-AUC
Valid	0.3488	0.8696	0.8884	0.8906	0.3436	0.8680	0.8919	0.8930	0.3478	0.8845	0.9256	0.9191
Speed bump	0.5799	0.7692	0.7757	0.9308	0.5354	0.7979	0.8204	0.9453	0.1220	0.8235	0.8389	0.9398
Lack of visibility	1.0698	0.3917	0.2998	0.7711	1.0863	0.3955	0.3051	0.7717	0.3160	0.4123	0.3596	0.7836
Bad parking spot	0.7848	0.6012	0.6268	0.8292	0.7753	0.6051	0.6332	0.8389	0.3940	0.6237	0.6566	0.8495
Traffic light	0.4237	0.7981	0.8305	0.9694	0.3727	0.8091	0.8551	0.9733	0.1176	0.8266	0.8908	0.9781
Stop line	0.5629	0.7473	0.8055	0.8262	0.5488	0.7534	0.8151	0.8355	0.5281	0.7084	0.7985	0.8092
Refuge island	0.6566	0.6852	0.7249	0.9186	0.6233	0.6963	0.7406	0.9257	0.2150	0.7464	0.7988	0.9527
Dangerous refuge island	0.8087	0.5798	0.5968	0.7395	0.8079	0.5404	0.5508	0.7053	0.5087	0.6160	0.6758	0.7788
Bus stop	0.8760	0.5214	0.4951	0.8909	0.7298	0.5588	0.5500	0.9104	0.0677	0.6185	0.5988	0.9440

#### 3.2.2 Task-specific results

**Speed bump detection**

The detection of speed bumps showed a significant variance in model performance, with ConvNeXtV2 exhibiting superior precision (PRC-AUC of 0.8389) compared to ResNet50 (0.7757) and ResNeXt50 (0.8204). This suggests ConvNeXtV2's enhanced ability to identify speed bump features from aerial images.

**Visibility and parking analysis**

Visibility issues presented a challenge across all models, with relatively lower PRC-AUC scores, indicating the difficulty of assessing visibility from images. Capturing low visibility scenarios was challenging both during the manual annotation process and in model training. The assessment of visibility issues is inherently subjective, as it requires annotators to judge whether certain elements obstruct the line of sight between pedestrians and drivers sufficiently to constitute a safety hazard. These obstructions can vary widely and include parked vehicles, vegetation, street furniture, signage, and temporary obstacles like construction equipment or waste containers. The diversity and complexity of these elements made it difficult to establish consistent criteria for annotation.

During manual annotation, annotators often faced difficulties in consistently identifying and labeling visibility obstructions. This subjectivity led to inconsistencies in the annotated data, which in turn affected the quality of the training data for the models. As a result, the models struggled to accurately identify obstructions or factors that impede driver-pedestrian visibility. Indeed, as shown in Fig. 10, the model incorrectly predicts a high probability of lack of visibility, likely interpreting the nearby trees as a sight obstruction when, in practice, they do not significantly obstruct driver-pedestrian visibility. The variability in environmental conditions and the subtlety of some obstructions further compounded the challenge.

This experience highlighted the need for a more extensive and diverse dataset with well-annotated examples of visibility issues. Improving the annotation process by providing clearer guidelines and training for annotators, possibly involving multiple annotators to reach consensus, could enhance the consistency and quality of the dataset. Additionally, incorporating more examples of various visibility obstructions would provide the models with better training data, potentially leading to improved performance in detecting low visibility scenarios.

ConvNeXtV2, however, demonstrated a modest improvement in performance. The analysis of bad parking spots saw a similar trend, with ConvNeXtV2 showing a slight edge in model precision and accuracy.

**Traffic management features**

The evaluation of traffic lights and stop lines at traffic lights highlighted the effectiveness of ConvNeXtV2, which achieved the highest PRC-AUC scores of 0.8908 and 0.7985, respectively. These results underscore the model's capability in recognizing traffic management infrastructures that influence pedestrian safety. For instance, in detecting traffic lights at crossings, ConvNeXtV2 achieved a PRC-AUC of 0.8908 compared to 0.8305 (ResNet50) and 0.8551 (ResNeXt50). Fig. 10 illustrates a correctly identified traffic light. The model assigns a probability of 1.0 for the presence of a traffic light in this example, matching the ground truth.

**Refuge islands**

**Figure 9:**
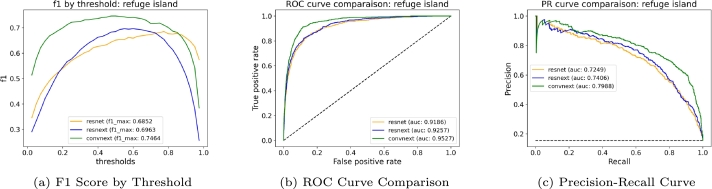
Comparative performance analysis of the models for the refuge islands. (a) F1 score across different thresholds, showing model sensitivity. (b) ROC curves, with AUC illustrating the models' true positive rate against the false positive rate. (c) Precision-Recall curves, with AUC scores indicating the models' precision-recall trade-off. The dashed line in (c) represents the performance of a naive classifier, i.e. a classifier that predicts the positive label in proportion to its frequency in the dataset.

The distinction between general and dangerous refuge islands was notably improved with ConvNeXtV2, which outperformed the other models in identifying potential risks associated with refuge island designs (Fig. 9).

**Figure 10:**
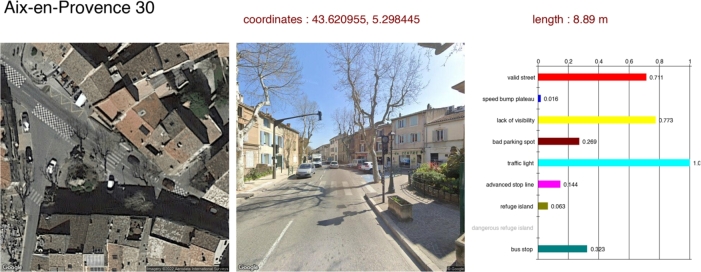
An example of the classification results for a zebra crossing. Left: the aerial image; center: the street view image; right: a graph with a value between 0 and 1 for each key variable. This example shows a correct recognition of a traffic light (presence of traffic light = 1.0) but an incorrect flag for lack of visibility, likely due to the presence of nearby trees that, in reality, do not significantly obstruct the driver's view.

**Bus stop proximity**

The analysis of bus stop proximity yielded varied results, with a significant imbalance in positive and negative labels affecting model training. Despite this, ConvNeXtV2 showed an improved ability to identify situations where bus stop placement could impact pedestrian safety, as indicated by a PRC-AUC score of 0.5988.

## 4 Discussion

The integration of advanced deep learning models, including ConvNeXtV2, ResNet50, and ResNeXt50, for the automated detection and classification of pedestrian crossings and roundabouts represents a significant advancement in road safety audits. The effectiveness of these models, particularly highlighted by the robust performance of the Mask R-CNN in detecting zebra crossings and roundabouts from aerial imagery, underscores their potential in enhancing pedestrian safety across diverse urban and rural settings. The ability of the ConvNeXtV2 model to outshine in the classification tasks, despite the data imbalance, further demonstrates the impact of architectural innovations in tackling complex image-based problems in road safety.

One notable challenge encountered was the models' struggle with instances of low visibility, a variable that is inherently difficult to capture due to its subjective nature and the variability in human annotation. This issue underscores the necessity for dataset augmentation with a wider range of visibility-impairing scenarios, aiming to provide a richer learning context for the models. Additionally, the imbalance in training data, especially for categories such as refuge islands and bus stop proximities, posed significant hurdles. However, the satisfactory classification results, enhanced by the incorporation of aerial images, indicate that varied perspectives in training data can ameliorate some of these challenges.

While our evaluation focused on convolutional neural network architectures like ResNet50, ResNeXt50, and ConvNeXtV2, novel architectures (e.g., Vision Transformers) could provide additional context. At the time of our analyses, transformer-based models for computer vision were still emerging and required substantial computational resources, which limited our ability to implement them. Future research could explore the application of Vision Transformers and other advanced architectures to potentially enhance performance further.

The application of segmentation techniques for measuring the length of zebra crossings and the proximity of roundabouts showcased promising scalability and objectivity, marking an improvement over manual, error-prone methods. Moreover, the utilization of Google Maps and Street View has been instrumental due to their comprehensive coverage and quality imaging, though the reliance on such platforms raises concerns about data availability and privacy across different regions. Coverage may vary geographically, with some areas lacking up-to-date or high-quality imagery, which can affect the model's ability to detect correctly the different variables. Additionally, changes in platform policies or access restrictions could limit future data collection and analysis efforts. There are also considerations regarding the ethical use of images that may include identifiable individuals or sensitive information. This highlights the importance of seeking adaptable and alternative data sources to ensure broader applicability of our approach.

In conclusion, our study illustrates the viability of leveraging advanced deep learning models for the critical task of road safety analysis. The challenges identified, including data imbalance and the complexity of capturing variables like visibility, point towards the need for continuous dataset enhancement and methodological refinement. Future research directions include:

• **Dataset Expansion and Balancing**: Collecting more data to balance the classes, particularly for underrepresented variables, and including more diverse environmental conditions to improve model robustness.
• **Advanced Modeling Techniques**: Exploring the use of transformer-based models or hybrid architectures, such as Vision Transformers, to enhance feature extraction and capture complex relationships.

These efforts aim to address current limitations and enhance the models' adaptability to diverse geographic contexts. As we progress, the adaptability of these models to diverse geographic contexts and the iterative improvement through recursive learning will be pivotal in paving the way for more effective and informed road safety interventions worldwide.

## CRediT authorship contribution statement

**Dylan Russon:** Writing – original draft, Visualization, Validation, Software, Methodology, Data curation. **Antoine Guennec:** Writing – review & editing, Validation, Software, Methodology, Data curation. **Juan Naredo-Turrado:** Writing – review & editing, Methodology, Investigation, Data curation, Conceptualization. **Binbin Xu:** Writing – review & editing, Validation, Investigation, Formal analysis, Data curation, Conceptualization. **Cédric Boussuge:** Writing – review & editing, Methodology, Formal analysis, Data curation, Conceptualization. **Valérie Battaglia:** Writing – review & editing, Supervision, Project administration, Investigation, Formal analysis, Conceptualization. **Benoit Hiron:** Supervision, Project administration, Funding acquisition, Conceptualization. **Emmanuel Lagarde:** Writing – review & editing, Validation, Supervision, Project administration, Methodology, Investigation, Funding acquisition, Formal analysis, Conceptualization.

## Declaration of Competing Interest

The authors declare that there is no conflict of interest.

## Figures and Tables

**Table A.4:** Comparison of results for models trained with only Street View images and models trained with both aerial and Street View image pairs for each key variable, evaluated on loss, F1 score, PRC-AUC, and ROC-AUC using the ResNet50 architecture.

Task	Stree tView only				Aerial-Street View pair			
	Loss	F1	PRC-AUC	ROC-AUC	Loss	F1	PRC-AUC	ROC-AUC
Valid	0.3350	0.8714	0.8955	0.9032	0.3488	0.8696	0.8884	0.8906
Speed bump	0.8332	0.6209	0.6470	0.8956	0.5799	0.7692	0.7757	0.9308
Lack of visibility	1.0049	0.3860	0.2709	0.7857	1.0698	0.3917	0.2998	0.7711
Bad parking spot	0.8348	0.5538	0.5777	0.7954	0.7848	0.6012	0.6268	0.8292
Traffic light	0.4868	0.7796	0.8507	0.9497	0.4237	0.7981	0.8305	0.9694
Stop line	0.7184	0.6580	0.5877	0.6496	0.5629	0.7473	0.8055	0.8262
Refuge island	0.6737	0.6321	0.6520	0.9033	0.6566	0.6852	0.7249	0.9186
Dangerous refuge island	1.0316	0.4158	0.3205	0.6996	0.8087	0.5798	0.5968	0.7395
Bus stop	0.9694	0.4431	0.3081	0.8456	0.8760	0.5214	0.4951	0.8909

**Figure C.14:**
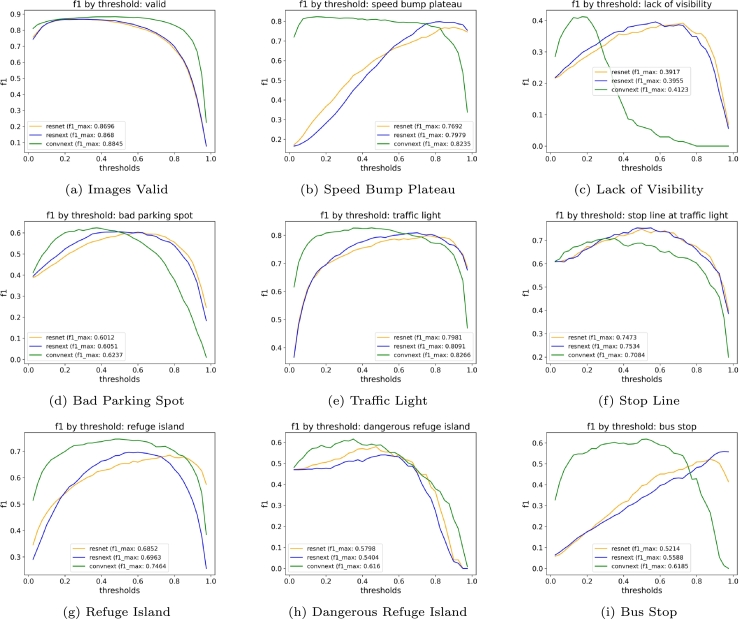
F1 Score by Threshold comparison for each key variables.

**Figure C.15:**
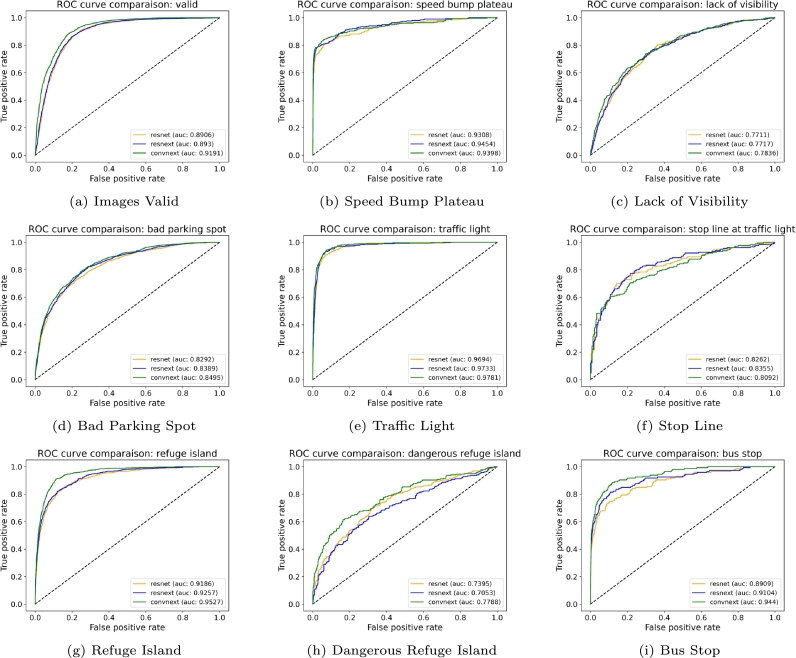
ROC curve by Threshold comparison for each key variables.

**Figure D.17:**
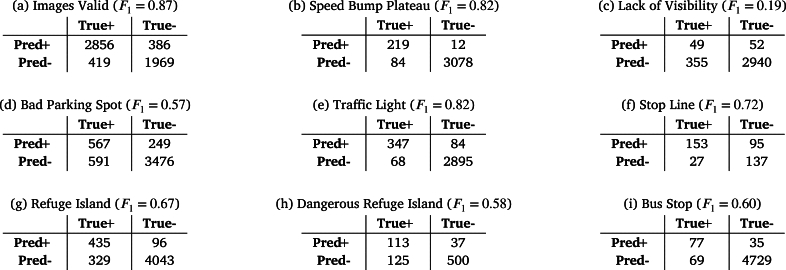
Confusion matrices for the nine tasks using ConvNeXtV2, arranged in a 3×3 grid. Here, each confusion matrix is evaluated at the threshold of 0.5.

## Appendix A Comparison of model performance using only street view images versus combined aerial and street view images

Table A.4 presents a detailed comparison of the model performance metrics for each key variable when trained using only Street View images versus when trained with both aerial and Street View image pairs, utilizing the ResNet50 architecture. Both models were trained on the same dataset distribution; however, the second model incorporated aerial images alongside the Street View images.

From the table, we see that including aerial images generally leads to improved performance across most tasks.

## Appendix C Classification metrics

Figure C.14, Figure C.15, Figure C.16 show F1 by threshold, ROC curves, and Precision-Recall curves for all key variables. The dashed lines in all plots in Fig. C.16 represent the naive baseline.

## Appendix D Confusion matrices for selected tasks

In this appendix, we present confusion matrices for each classification task for the ConvNeXtV2 model (see Fig. D.17). We use a threshold of 0.5 to obtain binary predictions. Each matrix shows the number of True Positives (TP), False Positives (FP), False Negatives (FN), and True Negatives (TN), providing insight into how the model performs for both the minority and majority classes.

## Footnotes

1 In comparison, the top models on the ImageNet benchmark have 250–550 million trainable parameters. See https://paperswithcode.com/sota/image-classification-on-imagenet.
